# Charting the Future of Oral Health: A Bibliometric Exploration of Quality-of-Life Research in Dentistry

**DOI:** 10.3390/ijerph21030249

**Published:** 2024-02-22

**Authors:** Agatha Ravi Vidiasratri, Lisdrianto Hanindriyo, Caroline Manuela Hartanto

**Affiliations:** 1 Department of Preventive and Community Dentistry, Faculty of Dentistry, Universitas Gadjah Mada, Yogyakarta 55281, Indonesia; lisdrianto_hanindriyo@ugm.ac.id; 2 Health Economics, Policy, and Management Programme, Department of Learning, Informatics, Management and Ethics (LIME), Karolinska Institutet, 171 77 Stockholm, Sweden; caroline.manuela.hartanto@stud.ki.se

**Keywords:** oral health quality of life, OHRQoL, bibliometric, VOS Viewer, Indonesia

## Abstract

In recent decades, the focus of health research has shifted to the impact of disease or impairment on how people proceed, behave, and experience quality of life. People’s lives are affected by oral diseases in various ways. Oral health-related quality of life (OHRQoL) is inextricably linked to general health and well-being, and it has far-reaching consequences for clinical practice and dentistry research. Particularly in Indonesia, increasing attention to OHRQoL is related to several concerning oral conditions, such as the extremely high number of cases of tooth decay and inflammation of dental supportive tissue that inexplicably lowers the population’s OHRQoL. To date, there has yet to be a bibliometric study of OHRQoL research in Indonesia. We intend to map the existing scientific literature on OHRQoL research in Indonesia during the last five years and investigate its research gaps. Scopus and the Sinta Database (a national database through Google Scholar) were used to retrieve Indonesian OHRQoL research publications from 2018 to 2023. Bibliographic data were analyzed using SPSS Statistics 25.0 and VOS Viewer 1.6.19. The data demonstrate that the number of OHRQoL-related publications in Indonesia and the number of local writers have increased over time. More of these publications were published in prestigious national journals than foreign ones. The study found that local researchers tended to conduct OHRQoL research on children and older populations, raising the issue of tooth decay or tooth loss. Exploring other subjects, such as dental anxiety, patient satisfaction, chewing performance, aesthetics, and appearance, and other populations (people with oral cancer and other systemic conditions) could broaden the environment of OHRQoL research in Indonesia.

## 1. Introduction

The approach in the healthcare system has evolved over time, shifting from a narrow focus—that targeted individual disease prevention—to a more comprehensive guide on human well-being [1,2]. In the past, health studies focused more on human mortality and morbidity. Nevertheless, the trend has changed over the years to focus on the impact of disease or impairment on how people proceed, behave, and perceive life quality [3,4]. Research on the importance of the biopsychosocial aspect in health has been growing for decades [5]. Life has multiple elements, including biopsychosocial elements, that will inevitably impact a person’s quality of life [6].

The World Health Organization (WHO) defines the quality of life as “individuals’ perceptions of their position in life in the context of the culture and systems in which they live, and about their goals, expectations, standards and concerns” [7]. It supports the definition of “health-related quality of life,” which involves multiple indicators in assessing health and treatment outcomes [8]. From the general health perceptions, the researchers elaborated a multidimensional approach to the quality of life that includes oral functional and psychosocial dimensions and created a separate model: “oral health-related quality of life (OHRQoL)” [9,10].

OHRQoL is an inseparable segment of overall health and well-being and has tremendous implications for clinical practice and dental research [11,12]. The first identified publication of OHRQoL papers was in 1994, and the trend has gradually increased since then [13]. The World Health Organization recognized that oral health is one of the determinant factors of human quality of life. The nongovernmental body wrote a separate chapter on OHRQoL in the World Oral Health Report 2003 [14]. The topic is relatively new [12]; however, the shift in the oral healthcare approach, focusing on patient-centered and biopsychosocial treatment, makes OHRQoL an essential part of the oral health research world.

Existing research identifies how OHRQoL contributes to survey research and measures as one of the variables in the analysis [10]. A previous descriptive paper shows that OHRQoL-related research worldwide has recently peaked in productivity [13]. There have been a few pieces of research on the topic in Indonesia, from clinical studies [15,16,17,18] to developing an instrument that fits a particular targeted population [19,20,21,22,23,24]. Growing attention to OHRQoL in Indonesia is related to some concerning conditions. The Indonesian National Health Survey 2018 shows that most Indonesians had not regularly visited a dentist in the past year even though their oral health conditions were concerning. Almost 90% of Indonesians suffer from tooth decay (caries), and more than 70% have gum disease (periodontitis) [25]. Moreover, the elderly population in Indonesia has been gradually increasing, from only 5% of the total population in 2010 to approximately 11% expected in 2035. Supporting this trend, the Indonesian life span also rose from 72.51 in 2015 to 75.47 in thirty years [26]. A longer life expectancy demands that people nurture their well-being for a better quality of life [27].

As one of the well-being indicators, OHRQoL can represent people’s quality of life from the oral health perspective [10]. Oral health may be associated with retained tooth conditions and dental treatment needs in older people with longer life spans [28,29]. The potential of oral health to affect people’s quality of life makes exploring OHRQoL topics in Indonesia critical in these times; thus, this bibliometric mapping was developed for that purpose [30,31].

Bibliometric mapping uses a scientific computed assistant to statistically evaluate published papers or books and explore high-impact research [32,33]. The assessment can explore the network between authors, institutions, and journals with a high number of contributions and analyze the keywords on the specific topic [13,33]. To date, there has been no bibliometric analysis of OHRQoL research in Indonesia.

Thus, we aim to (a) map the literature on OHRQoL in Indonesia during the last five years and (b) explore the research gap in Indonesian OHRQoL research. Our study addresses this gap by focusing on sociocultural influences in Indonesia, contributing globally by showcasing the country’s growing interest in OHRQoL. Additionally, our seek to encourage global collaboration, providing insights for cross-national studies. We also highlight the potential underrepresentation of local research in international databases, emphasizing the value of data in local publications for researchers worldwide.

## 2. Materials and Methods

### 2.1. Literature Search

In May 2023, an electronic literature search was undertaken using the Science and Technology Index (Sinta) via Google Scholar and Scopus. Sinta is a web-based database providing comprehensive research information in Indonesia [34]. A total sampling technique was used, and several sets of search terms were applied to both keyword engine searches and local and international databases. All document types of publications from 2018 to 2022 were retrieved, excluding thesis and dissertation papers. Only research conducted in Indonesia and only publications using Indonesian or English were included. Table 1 (Google Scholar) and Table 2 (Scopus) present the article selection strategy.

### 2.2. Data Extraction

Data were extracted using Publish or Perish (for the nationally published articles) and Scopus. Mendeley Web Importer and Mendeley Reference Manager were also used to retrieve and review the papers. The following bibliometric variables were extracted: citation information (authors, titles, publication year, citation), bibliographical information (affiliation, journal publisher, original document language), and abstract and keywords. Data were cleaned to eliminate duplicate and irrelevant papers based on the inclusion and exclusion criteria. Data extraction and cleaning were performed twice to prevent errors.

Extracting nationally published documents

The search results from Google Scholar in Publish or Perish were first sorted according to the document type and their relevance to the topic. The data were then exported to Microsoft Excel 16.78 for further duplicate data cleaning since the national database included some papers published by internationally accredited publishers. The selected articles were imported to Mendeley Research Manager using Mendeley Web Importer tools. The incomplete bibliographic information in Mendeley Research Manager was updated and then exported to RIS and Microsoft Excel 16.78 formats. We used the national journal indexation, the Science and Technology Index (Sinta) to rank the research papers; Sinta 1 is the highest rank, and Sinta 6 is the lowest rank.

Extracting internationally published documents

The search results from Scopus were reviewed and selected according to the research inclusion and exclusion criteria. The final results were saved to RIS and Microsoft Excel formats and exported to Mendeley Research Manager. We completed the missing information of bibliographic data in Mendeley to ensure proper referencing. We used the Scopus Indexation for the internationally published papers, with those in quartile 1 (Q1) at the top rank and quartile 4 (Q4) at the bottom.

### 2.3. Data Analysis

Information on the number of authors, citation count, study design, research focus, and OHRQoL instrument was summarized using a spreadsheet (Microsoft Excel 16.78). Other descriptive analyses on basic bibliometric information, such as the institution productivity, journal reputation, year of publication, and journal contributions, were input to and analyzed using SPSS Statistics 25.0. A co-occurrence map of OHRQoL high-frequency keywords was created using VOS Viewer 1.6.19. This application was also used to create a network visualization among the authors.

## 3. Results

After the literature search, data cleaning, and examination, 88 articles were included in the analysis. During data cleaning, several internationally accredited papers appeared in the both databases (Sinta/Google Scholar Database). Papers in the Google Scholar and Scopus databases were then sorted into two categories according to the journal accreditation: national and international. Table 3 shows the distribution of manuscripts on oral health-related quality of life (OHRQoL) and whether they were published in a national Indonesian or international journal. More than 60% of the output of OHRQoL research in Indonesia was published in locally accredited journals, while the rest received international recognition. The highest productivity of this topic in both journal categories was in 2022. The finding indicates a corresponding *p*-value of 0.685. These results suggest a lack of statistically significant association between the scope of publishers observed across the assessed years.

**Figure 1:**
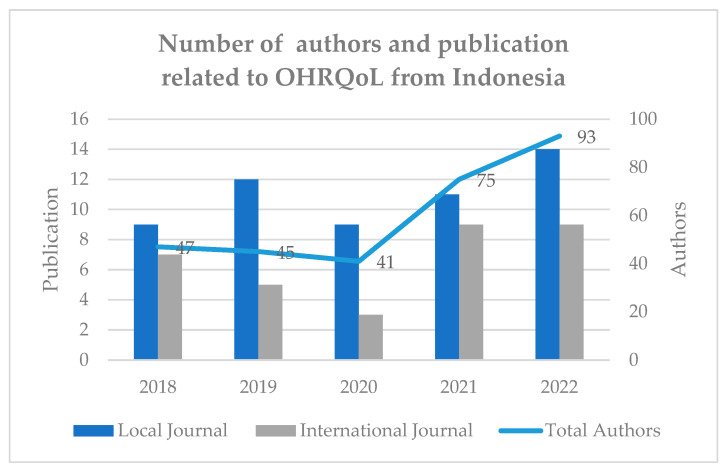
Number of disseminated Indonesian studies related to OHRQoL and the authors per year (N = 88).

Figure 1 illustrates the fluctuation of the number of total published papers over the research time periods in both databases. It dropped in 2020 but then bounced back in the next year and showed the highest publication number related to OHRQoL from Indonesia in 2022 in both local and international journals, although the number of research disseminators in local journals outnumbers the number of publications in international journals. Figure 1 also depicts the number of OHRQoL manuscript authors affiliated with Indonesia, which has increased over the years.

From Table 4, most Indonesian OHRQoL articles were published in reputable journals (Scopus or Sinta Journal Index); however, approximately 13% of the articles were published by local journals with no reputation. The manuscripts mostly passed the Q3 (third quartile) Scopus Rank and Sinta (Science and Technology Index) 3 National Rank among the reputed papers. Original articles dominated the overall journal type at more than 90%, followed by review articles, case reports, and proceedings.

Table 5 gives the list of Indonesian OHRQoL manuscripts obtaining the most citations during the period of the study, showing that the top two were systematic review papers from internationally reputed journals, with the highest number of citations per year for Bramantoro et al. (2021) [35]. The list also shows that the most common study design for original articles was a cross-sectional study, and the Oral Health Impact Profile (OHIP) was the most commonly used measure for assessing OHRQoL.

### 3.1. Contributing Journal and Collaboration Network

**Figure 2:**
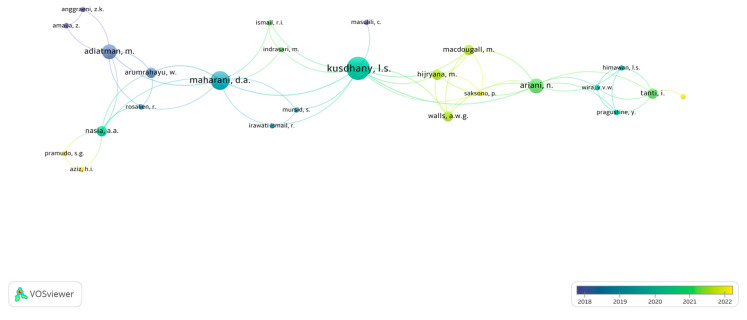
Collaboration of authors writing OHRQoL research papers (internationally accredited journals).

We evaluated contributing journals; overall, Universitas Indonesia is the most productive institution within this scope of research based on the number of articles, while Universitas Airlangga holds the highest number of citations per article (Table 6). Prolific authors Kusdhany, L.S.; Maharani, D.A.; Adiatman, M.; and Ariani, N. were networked, as seen in Figure 2. We also evaluated affiliation partnerships with 49 institutions from 9 countries that contributed to Indonesian OHRQoL research (Table 7).

The most common local journals publishing OHRQoL articles from 2018 to 2022 were *e-GiGi* (Impact Factor/IF = 0.945), *Makassar Dental Journal* (IF = 0.35), *and Jurnal Kedokteran Gigi Universitas Padjadjaran* (IF = 0), while the international journals were the *Journal of International Dental and Medical Research* (IF_2020_ = 0.26), which published more than 14% of Indonesia-affiliated OHRQoL research, and the *Journal of International Society of Preventive and Community Dentistry*.

### 3.2. Keyword Analysis

Nationally Accredited Journals

**Figure 3:**
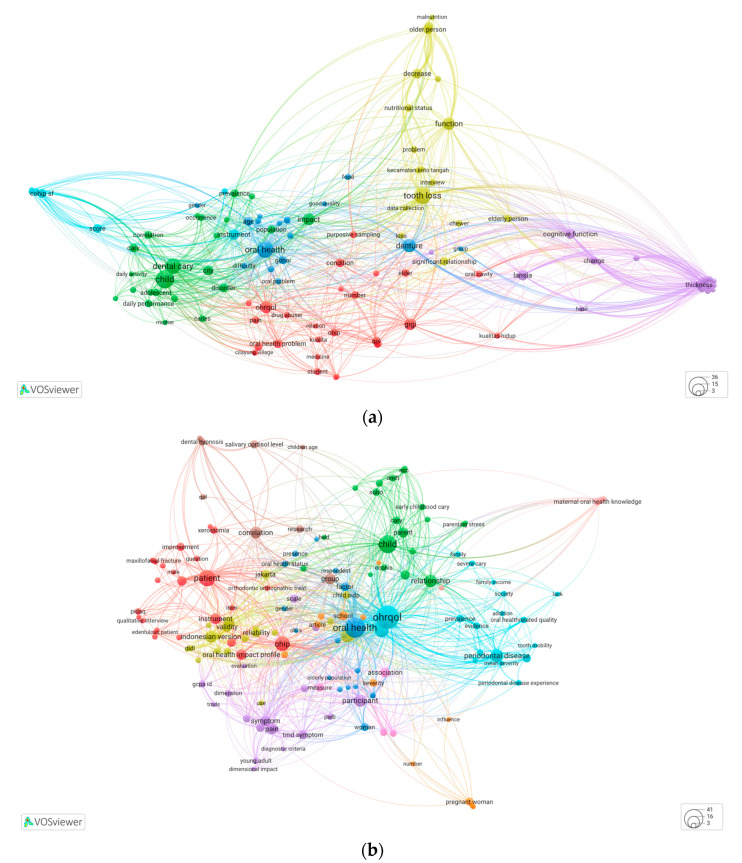
A co-occurrence map of Indonesian OHRQoL research, with colored bubbles indicating co-occurrence clusters and bubble sizes indicating the frequency of keyword occurrence: (**a**) Indonesian nationally accredited journals; (**b**) internationally accredited journals.

Analysis of the nationally reputed papers shows connections among 107 keywords with more than three occurrences, as illustrated in Figure 3a. Observing the high-frequency keywords for the target population, more authors are interested in research about children than those in the elderly and pregnant women. Keywords such as “dental caries” and “ECC” refer to early childhood caries, and “behaviour”, “nutritional status”, “pain”, “denture”, and “tooth loss” suggest high attention to prosthodontic, operative, and community dentistry. The most common instrument used in assessing OHRQoL in this research was the Oral Health Impact Profile (OHIP), followed by the Early Childhood Oral Health Impact Scale (ECOHIS), Geriatric Oral Health Assessment Index (GOHAI), and Child Oral Health Impact Profile—Short Form (COHIP-SF).

Figure 3a illustrates six different clusters of high-frequency keywords based on the research focus: cluster 1 (red): OHRQoL (27 items); cluster 2 (green): children and dental caries (23 items); cluster 3 (blue): denture and oral health (17 items); cluster 4 (yellow): tooth loss (17 items); cluster 5 (purple): elderly (16 items); and cluster 6 (light blue): instrument for OHRQoL assessment (7 items).

Internationally Accredited Journals

Figure 3b presents the connections among 151 keywords with more than three occurrences. For the target group, the authors of Indonesian OHRQoL research in the Scopus rank journals show quite a similar tendency to those in the local reputed journals, which targeted most school children. Other populations, such as the maternal age group, older people, and young adults, also frequently occur. High-frequency keywords related to instrument development, for instance, “reliability”, “validity”, “Cronbach”, “instrument”, “correlation”, “version”, and “reliability”, gained great attention. The most concerning oral conditions appeared in some clinically related keywords, such as “periodontal disease”, “xerostomia”, “ameloblastoma”, “maxillofacial fracture”, “maxillofacial trauma”, and “temporomandibular disorder”. Some keywords were associated with people’s subjective feelings, mental health, and perception toward dental treatments and conditions (“depression”, “dental anxiety”, “knowledge”, “psychosocial impact”).

A greater variety of OHRQoL assessment instruments were used among the Indonesian papers published internationally, including “Dental Impact on Daily Living/DIDL”, “Psychosocial Impact of Dental Aesthetics Questionnaire/PIDAQ”, “Graded Chronic Pain Scale/GCPS”, “Schizophrenia Health Outcomes/SHO”, “Child Oral Impact on Daily Performances/child OIDP”, and ECOHIS. The research focus in these journals was categorized into ten clusters, illustrated with different colors (Figure 3b): cluster 1 (red): oral health impact profile (25 items); cluster 2 (green): children and dental caries (20 items); cluster 3 (blue): elderly and oral health (20 items); cluster 4 (yellow): instrument for OHRQoL assessment (19 items); cluster 5 (purple): pain and temporomandibular disorder (19 items); cluster 6 (light blue): OHRQoL and periodontal disease (19 items); cluster 7 (orange): pregnant women (9 items); cluster 8 (brown): dental hypnosis (9 items); cluster 9 (pink): depression and drug (4 items); and cluster 10 (peach): dental health literacy.

## 4. Discussion

In mapping the existing scientific literature to oral health-related quality-of-life (OHRQoL) research in Indonesia, this study identified nearly one hundred articles related to OHRQoL for the past five years. The number of OHRQoL-related publications in Indonesia and the local authors fluctuated with a growing trend. The increasing pattern of publications in Indonesia is in agreement with the escalating global health research focus on psychosocial aspects [13,40,41,42]. The World Health Organization included social performance without pain and discomfort and mental and emotional well-being in their definition of oral health. Referring to oral health as a condition of teeth, mouth, and structures related to the mouth that allows people to carry out daily essential activities from eating, breathing, and speaking and to perform well socially leads to a more comprehensive outlook [43]. The goal of addressing oral health issues is not only to focus on treating a particular dental problem but also to take the overall well-being and the effort to enhance a patient’s daily functioning into account [13,44].

Despite the increase, the number of OHRQoL publications in Indonesia to date is limited. Health-related quality-of-life research was more common in nations or regions with higher GDP per capita since they had more established data systems, greater research funding, and epidemiological studies. Health-related quality of life is less frequently conducted in certain regions, such as Asia, South America, and Africa [41,45,46].

We also found that the number of OHRQoL-related manuscripts published in national journals outnumbered those published internationally. Local researchers tend to target national journal publishers due to the language requirements and research standards that seem more feasible. Researchers from English-speaking nations may have a particular advantage in explaining their findings and improving their papers’ acceptance since most international journals are published in English [47]. Countries with high levels of English proficiency showed more productivity and output from journal publishers with a good international reputation, especially in the medical field. The high number of publications in national journals may be related to the efforts of the Ministry of Science, Technology, and Higher Education of Indonesia in increasing publications within the country, which encourages academic students, professionals, and researchers to publish scientific articles in the Science and Technology Index/Sinta [48,49]. However, researchers should be aware that some national journals have not met the country’s standard, given that more than 10% of OHRQoL manuscripts in Indonesia are published without local or international accreditation.

The association between the scope of publishers and the years assessed of oral-health-related quality-of-life (OHRQoL) research in Indonesia was statistically insignificant. The absence of significance can be attributed to the limited number of current publications within the examined timeframe. This limitation may stem from various factors inherent to the research context. The field of OHRQoL research in Indonesia may still be in its developing stages [20,50], characterized by a relatively small pool of researchers and academic institutions actively engaged in producing scholarly outputs. Consequently, the volume of publications within a given period, such as the years assessed in this study, could be inherently constrained due to the developing nature of the field.

Cross-sectional study designs with an observational approach are the most frequently used in OHRQoL research in Indonesia. They are typically used to show an overview of a population’s traits, behaviors, outcomes, or factors associated with oral health [29,51]. Systematic review articles were among Indonesia’s most cited publications on OHRQoL, with 17.5 average citations per year. These articles explore the effects of clinical dental intervention [36] and dental community programs on people’s OHRQoL [35]. The systematic review provides a thorough overview of a particular area of research and recommendations for clinical practice and policy-making in the field of oral health [52].

The most frequent collaborations in conducting OHRQoL-related research in Indonesia were among researchers from institutions within the country. The second was with the neighboring country, Singapore, followed by other countries, such as the Netherlands, Malaysia, and the United Kingdom. Productive authors use connections and collaborations to their advantage, by which they could be the core of the collaboration network of the coauthor cluster [13,53]. Improving an author’s performance is often related to having robust grant funding, the allure of accredited academic institutions, and intensive communication between researchers [54,55].

During the last five years, the University of Indonesia has become the most productive institution in OHRQoL-related research. According to an international journal database, the authors affiliated with the University of Indonesia started exploring the topic of OHRQoL in 2011, earlier than other institutions in Indonesia that started approximately eight years later. The early start of OHRQoL research might build a robust basis for many more explorations in the field in the following years. In contrast, the University of Airlangga obtained the highest number of citations per year, despite its late start—compared to the University of Indonesia—in 2019. Their paper was a systematic review; thus, the result is in agreement with existing knowledge that review articles typically receive more citations than original research publications [56].

The mapping (Figure 2) illustrates the connections among the authors; the larger the bubble is, the more linkages and papers are published. As mentioned, the University of Indonesia produced the largest number of OHRQoL-related publications in the past five years, reflected in the authors’ mapping. The prolific authors, Kusdhany, L.S.; Maharani, D.A.; Adiatman, M.; and Ariani, N. are identified as affiliated with the University of Indonesia, having h-indexes ranging from 5 to 12.

The h-graph is one method of visualizing and comparing the productivity and effect of scholars’ published work. As the center of authors’ clusters, these prolific authors show a variety of collaborations with other authors within and outside their institutions. Although some research papers have initiated cooperation among institutions in Indonesia and abroad, most of the connection is still among the same faculty and university departments. More authors with high citations and h-indexes listed in the top 5 most cited OHRQoL publications did not appear in the mapping of the collaboration network. This might be because, in conducting the OHRQoL research, there was no collaboration between the institution and the University of Indonesia during the research period. These results may indicate that authors’ interdisciplinary and interinstitutional affiliations are still limited. Therefore, a more diverse and broader collaboration is encouraged to create a higher impact on the topic.

The top choices of national publishers among the Indonesian OHRQoL researchers were *e-GiGi* (IF = 0.945), *Makassar Dental Journal* (IF = 0.35), and *Jurnal Kedokteran Gigi Universitas Padjadjaran* (IF = 0). In contrast, the choice of the international publisher was the *Journal of International Dental and Medical Research* (IF_2020_ = 0.26). The impact factors range from 0 to below 1. These papers face difficulties in bringing notable benefits because of the low readability and potential impact on society. An impact factor calculates the typical number of citations each published paper in a journal receives [57,58]. It is a gauge for a journal’s readership; those with high impact factors are considered more reputable than those with low impact factors [59]. Comparing the top choices to the article obtaining the highest citations per year for OHRQoL research in Indonesia—that is, *PLoS One* (IF_2020_ = 3.240; average citations per year = 17.5)—the best course of action for the author might be to consider their choice of publisher for upcoming research on this particular topic.

Nevertheless, publishing in a national journal is also strategic for some topics aiming to deliver findings more pertinent to domestic populations. The national journal could improve the likelihood of its impact in particular geographic areas [58]. The impact shown by the number of citations determines whether an article was helpful for other authors but not for its benefit to people’s health [60].

We mapped the focus of the existing research on OHRQoL in Indonesia from the frequency of keyword appearance in bibliometric analysis. To obtain a larger picture from the frequent keywords, we divided the clusters into five categories: instrument development, targeted population, clinical conditions, dental promotion approach, and patient-centered subjective outcome measures. The previous article on worldwide OHRQoL research provides ground knowledge on the grouping, and the keywords in this research seem to fall into the same categorization as the worldwide findings [13]. However, some keywords regarding oral health promotion and prevention were detected in the national publishers, making adding the “dental promotion approach” as a new group necessary.

The term maps highlight keywords such as “child”, “tooth loss”, “denture”, “dental caries”, and “periodontal disease”. The larger bubbles show the choice of targeted population research based on age groups and clinical conditions, and oral treatments frequently appear in some articles. The past five years of OHRQoL publications have focused on children and older people. The popularity of research within these groups is aligned with the worldwide condition in which these two populations are most vulnerable to poor oral health [61,62,63]. Parameters such as certain age groups, relationship status, and history of chronic disease were found to have a substantial link with quality of life (QoL) [64]. The older a child experiences their first oral health examination, the higher the risk of them experiencing dental caries [61]. Tooth decay has been a concerning issue among children in Indonesia, with treatment being sought for their tooth problems in less than 2% of cases [65,66]. In addition, the rapid increase in the number of older people worldwide encourages the community to ensure proper access to preventive care and treatment for that particular population, as elderly individuals with tooth loss generally tend to suffer from poor health due to poor nutritional status [61,67]. The terms “prevalence” and “occurrence” create a cluster in keyword analysis among the articles published in the national publishers, which might show a higher acceptance rate for epidemiological studies in the publisher’s country.

To assess OHRQoL, researchers in Indonesia commonly use the Oral Health Impact Profile (OHIP) as the instrument. The pattern was presented as a rich connection between the keyword or its abbreviation and other terms. Some frequently cited OHRQoL articles in Indonesia used OHIP as an assessment instrument. Using the questions, OHIP could picture a broader wellness condition according to people’s perception of oral health [68]. OHIP has been translated into numerous languages and is available in short-form versions, which makes it more applicable in many situations [13]. Many more OHRQoL instruments are available across the world, for example, the Geriatric Oral Health Assessment Index (GOHAI), Oral Impacts on Daily Performance (OIDP), Dental Impacts on Daily Living (DIDL), and Early Childhood Oral Health Impact Scale (ECOHIS), which has been translated to an Indonesian version [21,69,70,71,72]. Using OHRQoL-specific instruments for a certain population is highly recommended to increase the robustness of the validity and reliability of an instrument.

The maps also picture the other OHRQoL research areas that have great potential to be explored since they build only small bubbles in the mapping visualization. Topics related to mental health (including stress), dental pain, or OHRQoL research are worth exploring more in Indonesia using a qualitative or mixed method (including interviews). Assessing the quality of life by interview can lead to a more comprehensive analysis of the process influencing people’s perception of quality of life [73]. When we compare the environment of OHRQoL research in Indonesia with other research worldwide [13,74], some areas seem not to have been explored yet in Indonesia: its relation with dental anxiety, patient satisfaction, chewing performance, aesthetics, and appearance and in other populations (people with oral cancer and any other systemic conditions). Adding these topics to future research plans could enrich OHRQoL research in Indonesia.

In both keyword maps, the term “OHRQoL” is not a prominent keyword compared to the others. Many authors did not include the word “OHRQoL” in their abstract. They used different terms to discuss this matter instead, for instance, by using the assessment instrument name (i.e., Oral Health Impact Profile). In another case, when the research mainly targeted children, the term often occurred as “ECOHIS” or “GOHAI” if the study targeted older people. In addition, the authors might use specific wordings related to the scope, the word “OHRQoL” or “oral health-related quality of life” in some cases detected as fragmented words instead of a single term. Some articles have also been published in Indonesian, and only a limited number of manuscripts are available for this research area, which may also contribute to the mapping outcome.

This study has other limitations, such as the keywords being limited to our experience-based knowledge and comprehension of OHRQoL, which is vulnerable to prejudice. We have applied as many queries as we observed from national and international databases; however, adding more questions in both languages that have yet to be covered could lead to more uncommon OHRQoL research in Indonesia. It is our understanding that the Sinta Database search engine does not allow users to export data recapitulation to other formats, either with or without a filter, complicating the screening process and making it more prone to error. During the research, we performed a double entry on our data and visual checking to reduce the risk of human error, such as duplicated publications or incomplete data. Double entry produced far fewer errors than only conducting visual checking or single entry [75].

We extracted documents published in the past five years, and more citations for the recently published articles might have been recorded since they were analyzed. According to research, it takes at least two to three years after publication for articles to accumulate enough sources for bibliometric indicators to be reliable [60,76]. However, to observe the recent environment of OHRQoL research in Indonesia, we documented the latest citation counts using Microsoft Excel 16.78 and analyzed the distribution using SPSS Statistics 25.0.

## 5. Conclusions

In conclusion, oral health-related quality-of-life (OHRQoL) research in Indonesia has gained significant traction in its continuous upwards trends, with studies exploring its association with various clinical conditions and dental community agendas. Both national and international publishers have contributed to a wide array of OHRQoL research areas involving diverse subject groups, with major focuses on OHRQoL concerning children, tooth loss, dentures, dental caries, and periodontal diseases. This research identified Universitas Indonesia, Universitas Padjajaran, and Universitas Airlangga as the leading institutions in OHRQoL research in Indonesia. In terms of journals, *e-GiGi*, *Makassar Dental Journal*, and *Jurnal Kedokteran Gigi Universitas Padjajaran* emerged as the top platforms for local publications, while *International Dental and Medical Research* and the *Journal of International Society of Preventive and Community Dentistry* stood out as prominent outlets for international dissemination of research findings.

While most manuscripts in Indonesia were published in national journals, fostering international collaborations, particularly with English-speaking nations, holds the potential to enhance the global visibility and impact of Indonesian research. This can be facilitated through collaborative initiatives and grant funding opportunities that promote interdisciplinary and interinstitutional affiliations. This study highlights existing research trends through bibliometric analyses and identifies areas for further exploration, such as mental health, dental pain, dental anxiety, patient satisfaction, chewing performance, and qualitative or mixed-method approaches. Researchers are encouraged to delve into these underexplored domains to enrich the existing body of knowledge and address the holistic well-being of diverse populations.

## Figures and Tables

**Table 1:** The flow of the literature search for Sinta through Google Scholar (search date: 1 May 2023).

Filter	Keyword Search (Indonesian–English)	Number of Hits
1	Query = (“kualitas hidup kesehatan gigi dan mulut” OR “oral health related quality of life” OR “OHRQoL” OR “OH-QoL” OR “OHRQL” OR “Oral Impact” OR “Oral Health Impact Profile” OR “Subjective Oral Health Status Indicators” OR “Dental Impact Profile” OR “children oral health profile” OR “early childhood oral health impact scale” OR “ECOHIS”) Publication Year: 2018 to 2022	990
2	Data cleaning: Title of publication, author, abstract, affiliation, publisher journal, relevance, document type	59
3	Data cleaning from inter-database duplication	57
4	Duplicate data cleaning within database	55

**Table 2:** The flow of the literature search for Scopus (search date: 1 May 2023).

Filter	Keyword Search (Indonesian–English)	Number of Hits
1	Query (English search terms) = (“oral health related quality of life” OR “OHRQoL” OR “OH-QoL” OR “ OHRQL” OR “Oral Impact” OR “Oral Health Impact Profile” OR “Subjective Oral Health Status Indicators” OR “Dental Impact Profile” OR “children oral health profile” OR “early childhood oral health impact scale” OR “ECOHIS”)	4888
2	Publication Year: 2018 to 2022	2545
3	Language: English, Indonesian Country/territory: Indonesia	39
4	The variables examined: title of publication, author, abstract, affiliation, publisher journal, relevance, document type	36
5	Data cleaning from inter-database duplication	33
6	Duplicate data cleaning within database	33

**Table 3:** Number of Indonesian studies related to OHRQoL published in Indonesian or international journals (N = 88).

Year	National		International		Total		*p*-Value *
	*n*	%	*n*	%	*n*	%	
2018	9	10.23	7	7.95	16	18.18	0.685 ^(ns)^
2019	12	13.64	5	5.68	17	19.32	
2020	9	10.23	3	3.41	12	13.64	
2021	11	12.50	9	10.23	20	22.73	
2022	14	15.91	9	10.23	23	26.14	
Total	55	62.50	33	37.50	88	100	

* Chi-square test; ^(ns)^ non-significant.

**Table 4:** Journal rank of Indonesian research dissemination related to OHRQoL year 2018–2022 (N = 88).

Domain	*n* (%)
**Journal Rank**	
Scopus Indexation (quartile)	
Q1	6 (17.65)
Q2	3 (8.82)
Q3	17 (50.00)
Q4	7 (20.59)
Indonesian Publication Indexation (Science and Technology Index/Sinta)	
Sinta 1	2 (3.64)
Sinta 2	7 (12.73)
Sinta 3	20 (36.36)
Sinta 4	13 (23.64)
Sinta 5	1 (1.82)
Sinta 6	0 (0.00)
No reputation	12 (21.82)
**Journal Type**	
Original articles	80 (89.9)
Review	5 (5.6)
Proceeding	1 (1.1)
Case report	3 (3.4)

**Table 5:** Information on the five most-cited OHRQoL publications in Indonesia.

No.	Title	Authors	Journal	Total Citations	Average Citations per Year	Study Design	Instrument
1	Outcomes of therapeutic TMD interventions on oral health related quality of life: A qualitative systematic review	Song Y.L., Yap A.U.-J., 2018 [36]	*Quintessence International*	36	7.2	Systematic review	Multiple instruments
2	Effectiveness of the school-based oral health promotion programmes from preschool to high school: A systematic review	Bramantoro T, Santoso C, Hariyani N et al., 2021 [35]	*PLoS ONE*	35	17.50	Systematic review	Multiple instruments
3	Effect of posterior tooth loss on the quality of life in the 45–65 years old (*Pengaruh kehilangan gigi posterior terhadap kualitas hidup pada kelompok usia 45–65 tahun)*	MN Rizkillah, RS Isnaeni, RPN Fadilah, 2019 [37]	*Padjadjaran Journal of Dental Researchers and Students*	25	6.25	Cross-sectional	OHIP
4	The relation of tooth caries and quality of life of school children aged 5–7 years old (*Hubungan karies gigi dengan kualitas hidup pada anak sekolah usia 5–7 tahun)*	B Nurwati, Setijanto, D., Budi, HS, 2019 [38]	*Jurnal Skala Kesehatan*	18	4.5	Cross-sectional	ECOHIS
5	The relation of tooth caries to quality of life of teenager in the city of Jambi (*Hubungan Karies Gigi dengan Kualitas Hidup Remaja SMA di Kota Jambi)*	H Boy, A Khairullah, 2019 [39]	*Jurnal Kesehatan Gigi*	16	4	Cross-sectional	OHIP

**Table 6:** Top three most productive OHRQoL-related research institutions in Indonesia.

Institution	Number of Articles (%)	Number of Citations	Citations per Article
Universitas Indonesia	18 (20.2)	82	4.56
Universitas Padjadjaran	10 (12.5)	33	3.30
Universitas Airlangga	6 (6.7)	59	9.83

**Table 7:** Countries participating in OHRQoL research (Scopus-accredited journals).

Country	Documents	Citations
Indonesia	33	110
Malaysia	2	37
Singapore	4	30
China	1	25
Hong Kong	1	25
Hungary	1	12
Netherlands	3	10
United Kingdom	2	5
Switzerland	1	4

## Data Availability

The data presented in this study are available in Scopus (scopus.com) and the Science and Technology Index/Sinta (sinta.kemendikbud.go.id.) website. The data supporting the conclusions of this article will be made available by the authors on request.
